# Construction and validation of a novel coagulation-related 7-gene prognostic signature for gastric cancer

**DOI:** 10.3389/fgene.2022.957655

**Published:** 2022-08-29

**Authors:** Bofang Wang, Dan Zou, Na Wang, Haotian Wang, Tao Zhang, Lei Gao, Chenhui Ma, Peng Zheng, Baohong Gu, Xuemei Li, Yunpeng Wang, Puyi He, Yanling Ma, Xueyan Wang, Hao Chen

**Affiliations:** ^1^ Second Clinical Medical College, Lanzhou University, Lanzhou, China; ^2^ Chengdu Seventh People’s Hospital, Chengdu, China; ^3^ State Key Laboratory of Genetic Resources and Evolution/Key Laboratory of Healthy Aging Research of Yunnan Province, Kunming Institute of Zoology, Chinese Academy of Sciences, Kunming, China; ^4^ Department of oncology, First Hospital of Lanzhou University, Lanzhou, China; ^5^ Key Laboratory of the Digestive System Tumors of Gansu Province, Lanzhou, China; ^6^ Department of Cancer Center, Lanzhou University Second Hospital, Lanzhou, China

**Keywords:** gastric cancer, coagulation-related genes, prognostic signature, weighted gene co-expression network analysis (WGCNA), bioinformatics

## Abstract

**Background:** Gastric cancer (GC) is the most common malignant tumor. Due to the lack of practical molecular markers, the prognosis of patients with advanced gastric cancer is still poor. A number of studies have confirmed that the coagulation system is closely related to tumor progression. Therefore, the purpose of this study was to construct a coagulation-related gene signature and prognostic model for GC by bioinformatics methods.

**Methods:** We downloaded the gene expression and clinical data of GC patients from the TCGA and GEO databases. In total, 216 coagulation-related genes (CRGs) were obtained from AmiGO 2. Weighted gene co-expression network analysis (WGCNA) was used to identify coagulation-related genes associated with the clinical features of GC. Last absolute shrinkage and selection operator (LASSO) Cox regression was utilized to shrink the relevant predictors of the coagulation system, and a Coag-Score prognostic model was constructed based on the coefficients. According to this risk model, GC patients were divided into high-risk and low-risk groups, and overall survival (OS) curves and receiver operating characteristic (ROC) curves were drawn in the training and validation sets, respectively. We also constructed nomograms for predicting 1-, 2-, and 3-year survival in GC patients. Single-sample gene set enrichment analysis (ssGSEA) was exploited to explore immune cells’ underlying mechanisms and correlations. The expression levels of coagulation-related genes were verified by real-time quantitative polymerase chain reaction (qRT-PCR) and immunohistochemistry (IHC).

**Results:** We identified seven CRGs employed to construct a Coag-Score risk model using WGCNA combined with LASSO regression. In both training and validation sets, GC patients in the high-risk group had worse OS than those in the low-risk group, and Coag-Score was identified as an independent predictor of OS, and the nomogram provided a quantitative method to predict the 1-, 2-, and 3-year survival rates of GC patients. Functional analysis showed that Coag-Score was mainly related to the MAPK signaling pathway, complement and coagulation cascades, angiogenesis, epithelial–mesenchymal transition (EMT), and KRAS signaling pathway. In addition, the high-risk group had a significantly higher infiltration enrichment score and was positively associated with immune checkpoint gene expression. **Conclusion:** Coagulation-related gene models provide new insights and targets for the diagnosis, prognosis prediction, and treatment management of GC patients.

## Introduction

Gastric cancer (GC) is the fifth most common cancer and the third leading cause of cancer death worldwide, with approximately one million new cases of GC every year, and about 784,000 patients died of GC (Smyth et al., 2020). The current detection methods are limited, resulting in a low diagnosis rate of early GC. Most patients have advanced GC when they are diagnosed. The prognosis of patients with advanced GC is abysmal, and the 5-year survival rate is less than 30% (Sung et al., 2021). With the aging of the social population, the incidence and mortality of GC continue to increase year by year, and tumor metastasis is the leading cause of high mortality. Cancer invasion and metastasis is a complex process controlled by multiple molecular determinants, which may involve activation of oncogenes, inactivation of tumor suppressor genes, and abnormalities in related signaling pathways (Hanahan and Weinberg, 2000). Biomarkers developed for key molecules play an essential role in the diagnosis, prognosis prediction, and the selection of treatment strategies for GC.

Traditional biomarkers such as CEA and CA19-9 lack sufficient specificity and sensitivity in current clinical applications. Drugs targeting Her-2 significantly prolong survival in patients with Her-2-positive GC, but their prognostic and predictive value performance remains ambiguous (Xiao and Zhou, 2017; Matsuoka and Yashiro, 2018). Therefore, the development of novel and effective GC biomarkers is necessary.

Venous thromboembolism (VTE) is often an underlying clinical symptom of cancer, and it remains one of the leading causes of cancer-related morbidity and mortality. Many studies have proved that patients with malignant tumors are in hypercoagulable and hyperfibrinolytic states, and the disorder of the coagulation system is related to tumor progression and prognosis (Repetto and De Re, 2017). The presence of tumors may strongly influence host coagulation and hemostasis systems by altering the molecular context to promote tumor cell growth, progression, and metastasis. Tumor-driven coagulation pathway activation leads to increased FGB of Fibrinopeptide A (FpA), accompanied by fibrin lysis and D-dimer (DD) release. Blockade of coagulation, fibrinolysis, and platelet activation pathways can effectively prevent tumor progression (Repetto and De Re, 2017). Numerous individual coagulation-related biomarkers revealed correlations with prognosis prediction in GC. Many laboratory data and clinical studies have shown that coagulation-related factors, such as tissue factor (TF), thrombin, plasminogen (PLG), FpA, DDs, TAFI, and thrombin–antithrombin complex, are involved in angiogenesis, tumor cell invasion, tumor progression, and metastasis (Dupuy et al., 2003; Buller et al., 2007). Coagulation-related factors are considered as diagnostic and therapeutic evaluation tools for thrombosis in patients with GC and are also regarded as independent factors and indicators to predict the prognosis of GC (Tas et al., 2013). A retrospective clinical study showed that rivaroxaban, a coagulation factor-targeted drug, could increase the efficacy of immune checkpoint inhibitors (ICIs) by restoring host antitumor immunity (Haist et al., 2021). Therefore, we hypothesized that coagulation-related biomarkers play a crucial role in evaluating the prognosis of GC. As far as we know, there are few studies on coagulation-related genes and the prognosis of GC.

**FIGURE 1:**
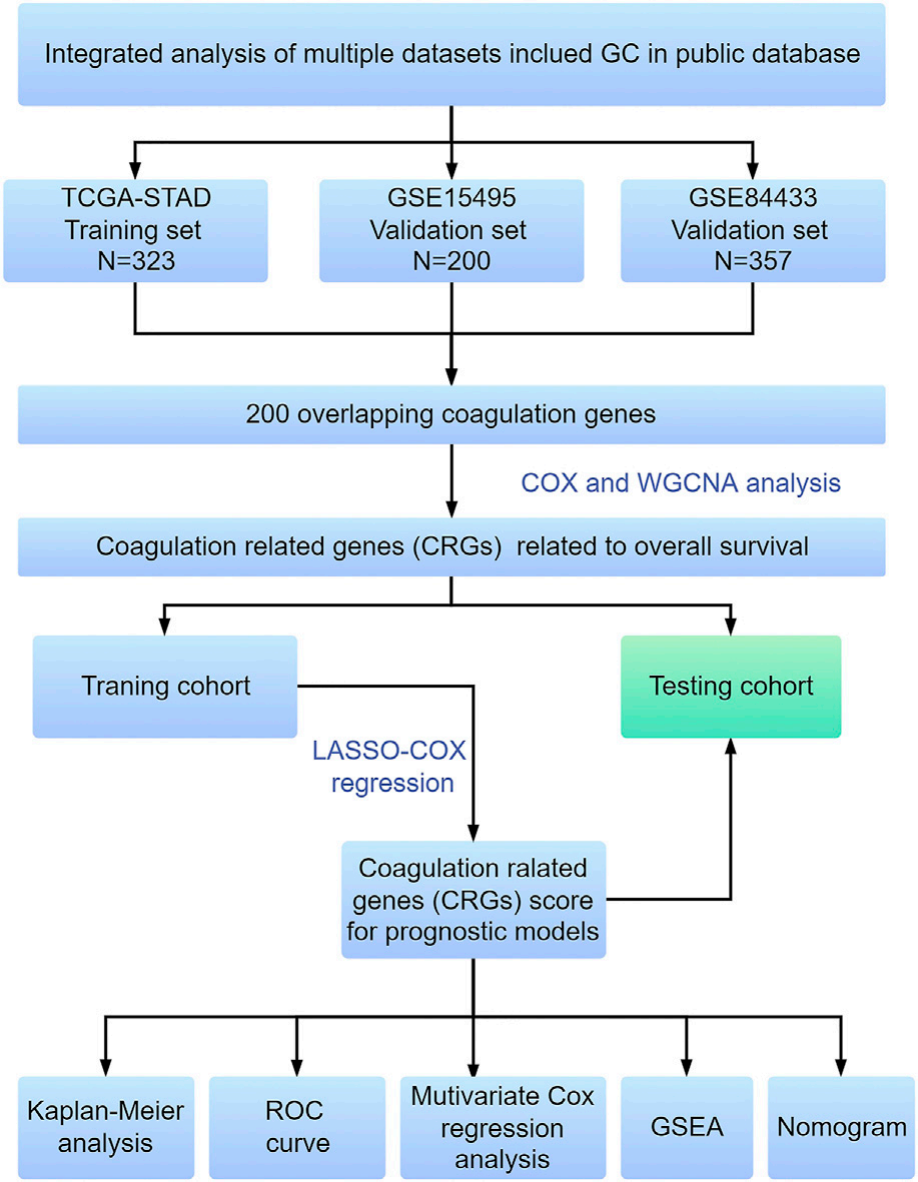
Flowchart of experimental design and main procedures.

In this study, we identified coagulation-related genes (CRGs) associated with the clinical features of GC through weighted gene co-expression network analysis (WGCNA), integrated expression profiles, and clinical information from multiple datasets of TCGA and GEO databases. We constructed a risk-score model based on seven CRGs, which provided a new model for accurately predicting the prognosis and individualized treatment of GC patients. The current research workflow is shown in Figure 1.

## Methods

### Data acquisition

We obtained the RNA expression data and clinical data of GC samples from the UCSC Xena database (http://xena.ucsc.edu/) based on the Cancer Genome Atlas (TCGA) and Gene Expression Omnibus (GEO) database (https://www.ncbi.nlm.nih.gov/geo/). The GEO database included the GSE15459 dataset and GSE84433 dataset. The TCGA-STAD dataset, GSE15459 dataset, and GSE84433 dataset included 323, 200, and 357 tumor samples, respectively. According to the annotation file provided by the platform, we used the mean to represent the expression level for a gene containing multiple probes.

### Coagulation-related co-expression network construction by WGCNA

AmiGO 22 is a web-based set of tools for searching and browsing gene ontology databases (http://amigo.geneontology.org/amigo). We obtained 216 gene symbols related to coagulation from AmiGO 2. Then 200 CRGs were obtained through the comprehensive analysis of TCGA-STAD, GSE15495, and GSE84433 datasets. To calculate the Z-score of the clinical information of the TCGA-STAD dataset, we used the “WGCNA” package in the R software to perform a WGCNA on the CRGs in the TCGA-STAD dataset (Langfelder and Horvath, 2008). The operational process of WGCNA includes cluster analysis of expression profiles and calculation of associations between each cluster module and clinical phenotypes. We used the soft threshold method for the Pearson correlation analysis of the expression profiles to construct a weighted network. In this study, the hub threshold was set to 0.9 and the minimum number of modules was set to 15. A cluster dendrogram was used to display the results of gene merging and classification. Finally, we analyzed the relationship between MEs and clinical traits, and identified the relevant modules. CRGs in modules associated with clinical features were selected as candidate genes.

### Coag-Score model construction

LASSO regression is a compression estimation method. By constructing a penalty function, the variable coefficients can be compressed so that the regression coefficients of some variables become 0 to achieve the purpose of screening variables (Wang et al., 2020). The “glmnet” package was used in the R software; we applied the LASSO regression method to construct a Coag-Score model of CRGs with optimal weighted coefficients. The CV curve was further drawn, the cross coefficient λ was derived using the cross-validation method, and the λ value with the best cross-validation error was selected. The regression coefficient of the best model was extracted to fit the new model. The final Coag-Score was calculated based on the expression of the gene multiplied by the corresponding regression coefficient. Based on the expression of genes in the model, PCA was carried out with the “prcomp” function of the “stats” R package.

### Coag-Score model verification

First, the Coag-Score of each sample in the TCGA-STAD dataset was calculated, and the patients were categorized into high-risk and low-risk groups according to the cutoff value; the constructed Coag-Score model was verified internally. We used the “Survival” and “KMsurv” packages in R software to conduct the Kaplan–Meier (KM) survival analysis and draw survival curves. We integrated the GSE15495 and GSE84433 datasets into the GEO cohort. External validation of the Coag-Score model was performed using the GEO cohort validation dataset. Receiver operating characteristic (ROC) curves were used to evaluate the Coag-Score’s accuracy and predictive power using the “survivalROC” package.

### Gene set enrichment analysis

Enrichment analysis between the high-risk and low-risk groups of GC patients was performed by Gene Set Enrichment Analysis (GSEA, https://www.gsea-msigdb.org/gsea/index.jsp) v4.3.1 software (Mootha et al., 2003; Subramanian et al., 2005). We selected the KEGG and HALLMARK gene sets for GSEA. Permutation testing (1000 permutations) was used to calculate enrichment scores (ES) and normalized enrichment scores (NES). NES with *p* values <0.05 and FDR <25% were considered to be significantly enriched.

### Construction and assessment nomogram for GC patients

A predictive nomogram of GC patients was constructed using the TCGA-STAD training set variables. The nomogram was constructed by using the “survival” and “RMS” packages, and Harrell’s Concordance Index (C-index) was used to estimate the prognostic effect of the prediction model.

### Correlation of immune cell infiltration and immune checkpoint gene expression with Coag-Score

Single-sample gene set enrichment analysis (ssGSEA) in the “GSVA” R package was used to quantify the infiltration level of 16 immune cells in each GC patient (Zuo et al., 2020). The TIMER database (http://timer.cistrome.org/) was used to analyze the correlation between Coag-score and immune cells. Moreover, the “ggpubr” package was used to plot violins to describe the correlation between Coag-Score and immune checkpoint gene expression.

### Tissue samples

In this study, paired GC tissues and adjacent non-cancer tissues were acquired from the Lanzhou University Second Hospital. Cohort 1: ten pairs of fresh GC tissues and adjacent non-cancer tissues were cryopreserved and were used for the quantitative analysis of the expression of CRG mRNAs by qRT-PCR. Cohort 2: 84 pairs of GC tissues and adjacent non-cancer tissues were formalin-fixed and paraffin-embedded, which were used to detect the expression of SERPINE1 protein in GC and adjacent tissues by immunohistochemistry (IHC). All patients signed an informed consent form. The Ethics Committee approved the study of Lanzhou University Second Hospital (Ethical Application Ref: 2019A-321).

### qRT-PCR

The total RNA of GC tissues and adjacent non-cancer tissues was extracted using TRIzol reagent (Thermo Scientific, United States) according to the product instructions. cDNA synthesis was performed using the PrimeScript™ RT reagent kit (Takara, Japan). qRT-PCR was performed using the SYBR Primix Ex Taq™ II (Takara, Japan) on ABI-7500 instrument (Applied Biosystems, United States). GAPDH was used as an internal reference gene, and the 2^-△△Ct^ method was used to compare the differential expression of genes. The primers are listed in Supplementary Table S1.

### Immunohistochemical staining

Immunohistochemical staining was performed according to the standard procedure. GC tissues were embedded into wax blocks, and then sections were prepared, which were dewaxed in xylene, and hydrated in gradient concentration alcohol, which were then washed with PBS buffer. Antigen repair was performed with citrate buffer in a water bath. Then the sections were incubated with the primary antibody anti-mouse SERPINE1 (1:200, abcam) overnight at 4°C. The sections were washed with PBS the next day and incubated with the secondary antibody for 60 min at room temperature. Then, the DAB kit (MXB biotechnologies, Fujian, China) was used to stain tissue samples.

After staining of GC tissue, an IHC score was performed by pathologists. According to the proportion of positive cells and staining intensity, the scores were divided as follows: 0 was negatively stained or <5% positive cells, one was weakly stained or 6–25% positive cells, two was moderately stained or 26–50% positive cells, and three was strongly positive or >50% positive cells. We defined the final staining score ≥3 as a high expression of SERPINE1, and the patients were divided into the high expression group and low expression group based on this staining score.

### Statistical analysis

Data were presented as mean ± SEM. We used the Student’s t-test to examine the difference in mean between the two groups. Non-abnormal distribution data were analyzed using a nonparametric test. The Kaplan–Meier method was used to compare the survival times of different CRG expression levels. Univariate Cox regression analysis was used to analyze the prognostic value of a single gene; for factors with a *p*-value < 0.05, we performed multivariate Cox regression to analyze the independent risk factors that affect GC patients. *p*-value <0.05 was considered to be statistically different. * represents *p* < 0.05 and ** represents *p* < 0.01. Statistical analysis was performed using the SPSS software package (version 24.0, IBM SPSS) and GraphPad Prism (version 8.0, GraphPad Software).

## Results

### Defining CRGs associated with clinical features

**FIGURE 2:**
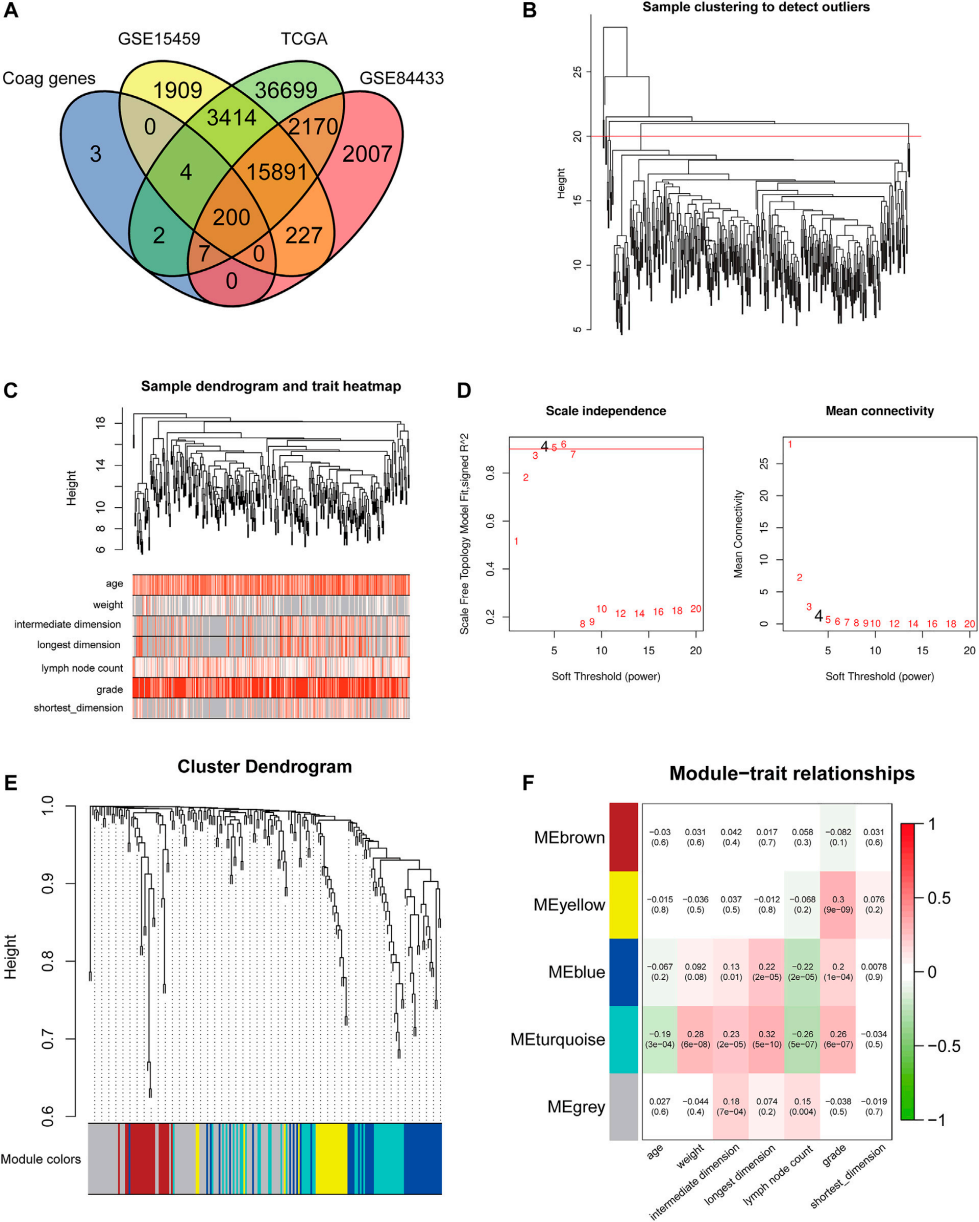
Construction of the co-expression modules of coagulation genes related to clinical characteristics of GC. **(A)** Venn diagram of CRGs in GC. **(B)** Sample clustering of CRGs. **(C)** Sample dendrogram and corresponding clinical traits. **(D)** The soft threshold of the CRG module is defined by scale independence and mean connectivity. **(E)** Correlation between sample clustering and modules. **(F)** The relationship between CRG module and clinical features of GC.

A total of 974 GC patients from five cohorts were included in this study, including the training set TCGA-STAD, the validation sets GSE15495 and GSE84433, cohort 1, and cohort 2 from the Department of Oncology of Lanzhou University Second Hospital. CRGs were obtained from the AmiGO 2 database and compared with TCGA-STAD, GSE15495, and GSE84433 datasets. A total of 200 overlapping CRGs were selected (Figure 2A).

To better understand the gene expression network during GC development, we used WGCNA to construct co-expression networks and identify co-expression modules associated with clinical features. The hierarchical clustering method was first used to obtain the GC sample cluster diagram, and the outlier samples were eliminated (Figure 2B). We analyzed the sample dendrogram and corresponding clinical traits (Figure 2C). The network analysis was performed, and an appropriate adjacency matrix weight parameter β was selected to satisfy the scale-free distribution as much as possible. We determined the appropriate soft threshold from the scale-free topology model fit-R^2^. We selected β from the first approach of 0.09 to construct the gene module (*β* = 4) and divided CRGs into five modules (Figures 2D,E). After obtaining different gene modules, a correlation analysis was conducted between the clinical features of GC and the module eigengene (ME) value of each module. Among them, the genes in the blue and turquoise modules were highly correlated with the age, weight, tumor size, the number of lymph nodes, and tumor grade of GC patients (Figure 2F). Therefore, the blue and turquoise module genes were selected for further analysis. The blue module contains 87 CRGs, and the turquoise module contains 54 CRGs.

### Construction of Coag-Score prognostic model based on CRGs associated with clinical features

**FIGURE 3:**
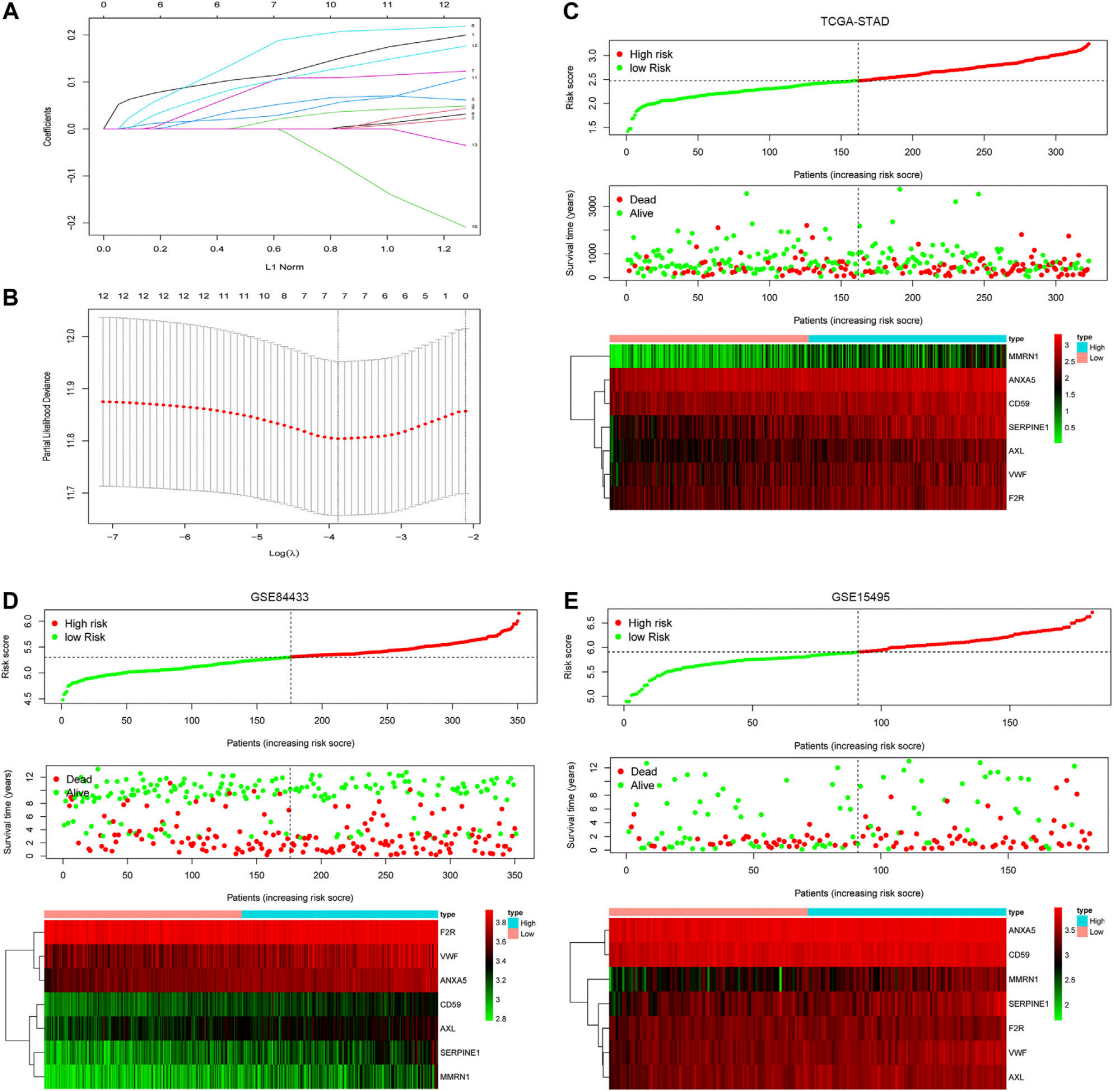
Coag-Score model construction. **(A,B)** Seven CRGs were screened based on LASSO regression analysis. **(C–E)** The distributions of the risk score for each patient (top panel), survival status of patients (middle panel), heatmaps for seven-gene signature between high-risk group and low-risk group (bottom panel).

In order to construct a simple and effective prognostic model, the LASSO Cox regression analysis was used to reduce the dimension of CRGs further. Seven CRGs were included in the model: Serpin Family E Member 1 (SERPINE1), von Willebrand factor (VWF), Coagulation Factor II Thrombin Receptor (F2R), Annexin A5 (ANXA5), CD59, AXL Receptor Tyrosine Kinase (AXL), and Multimerin 1 (MMRN1) (Figures 3A,B). The hazard ratios of SERPINE1, VWF, F2R, ANXA5, CD59, AXL, and MMRN1 were 1.234, 1.274, 1.303, 1.456, 1.403, 1.312, and 1.298 (*p* < 0.05), respectively. Based on the expression values and correlation coefficients of these seven CRGs, the prognostic risk score of each GC patient sample was calculated. Coag-Score = (0.110×expression level of SERPINE1) + (0.012×expression level of VWF) + (0.0460×expression level of F2R) + (0.167×expression level of ANXA5) + (0.092×expression level of CD59) + (0.026×expression level of ANXA5) + (0.097×expression level of MMRN1). GC patients were divided into high-risk and low-risk groups according to Coag-Score. Figures 3C–E show the risk curves of high-risk and low-risk groups (top panel), survival status (middle panel), and heatmap of single gene expression (bottom panel). The PCA indicated the patients in different risk groups were distributed in two directions (Supplementary Figure S1A-C).

### Comparison of prognostic models in the training set and the validation set

**FIGURE 4:**
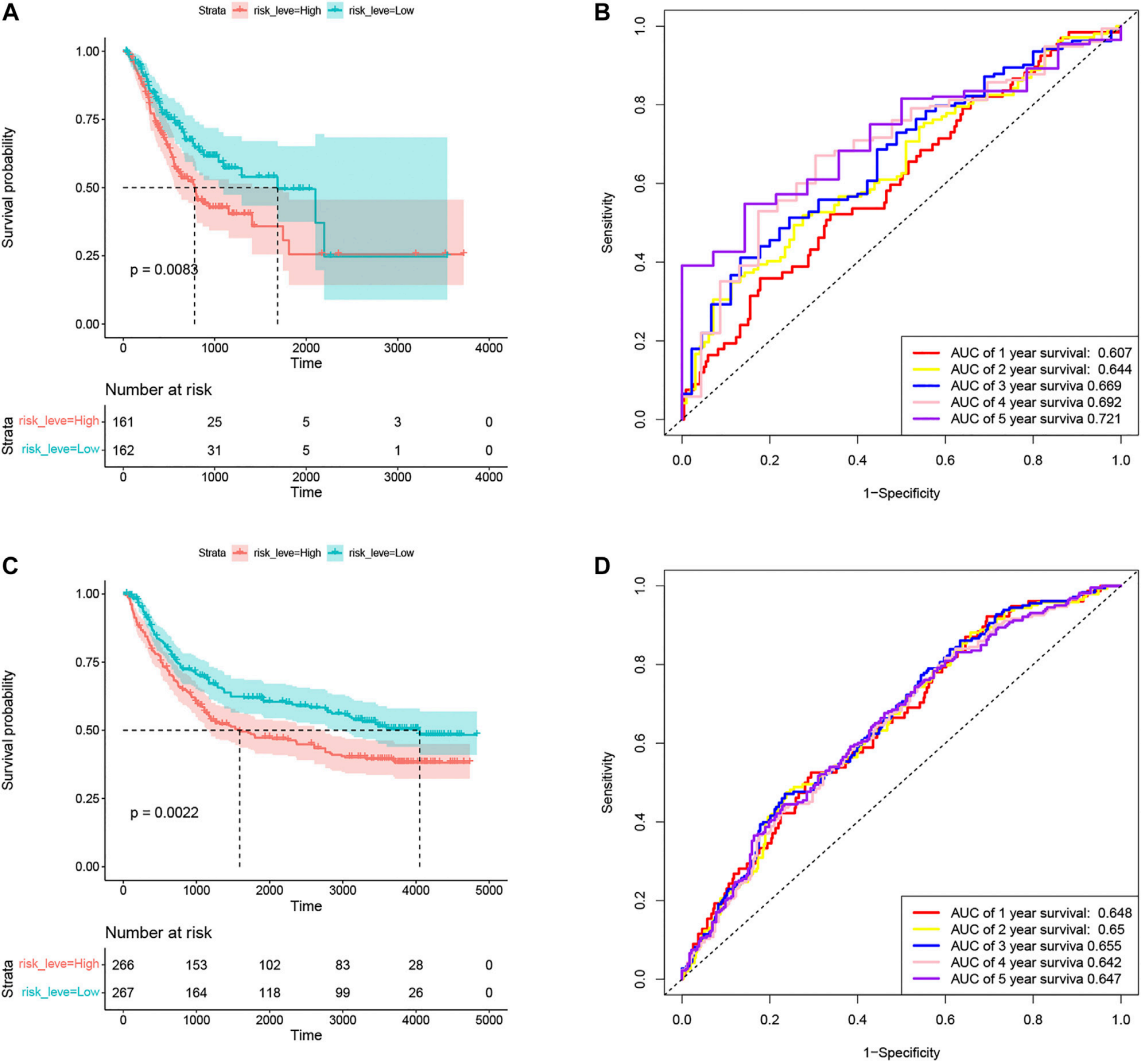
Evaluation and validation of the prognostic performance of Coag-Score in training and validation sets. **(A,C)** KM survival curves of Coag-Score in TCGA-STAD and GEO cohort. **(B,D)** ROC curves of Coag-score in TCGA-STAD and GEO cohort.

In the training set TCGA-STAD, compared with patients in the low-risk group, the OS of GC patients in the high-risk group was significantly lower (Figure 4A). In order to verify the prediction performance of Coag-Score on different datasets, GSE15495 and GSE84433 datasets were used as validation sets. We integrated GSE15495 and GSE84433 datasets into the GEO cohort for analysis. The validation set survival analysis was consistent with the training set TCGA-STAD results (Figure 4C). The log-rank p values of the KM curve were 0.0083 and 0.0022, respectively. ROC curves were used to evaluate the sensitivity and specificity of the Coag-Score signature for the prognosis of GC patients (Figures 4B,D). The results show that the areas under the curve (AUCs) of the training set TCGA-STAD 1, 2, 3, 4, and 5 years were 0.607, 0.644, 0.669, 0.692, and 0.721, respectively. The AUCs of the validation set were 0.648, 0.65, 0.655, 0.642, and 0.647, respectively. Univariate and multivariate Cox regression analyses showed the Coag-Score was an independent risk factor affecting the prognosis of GC patients, and the Coag-Score had better predictive power and accuracy than other prognosis-related metrics (Supplementary Figure S2A-E).

### Construction of nomogram for GC patients

**FIGURE 5:**
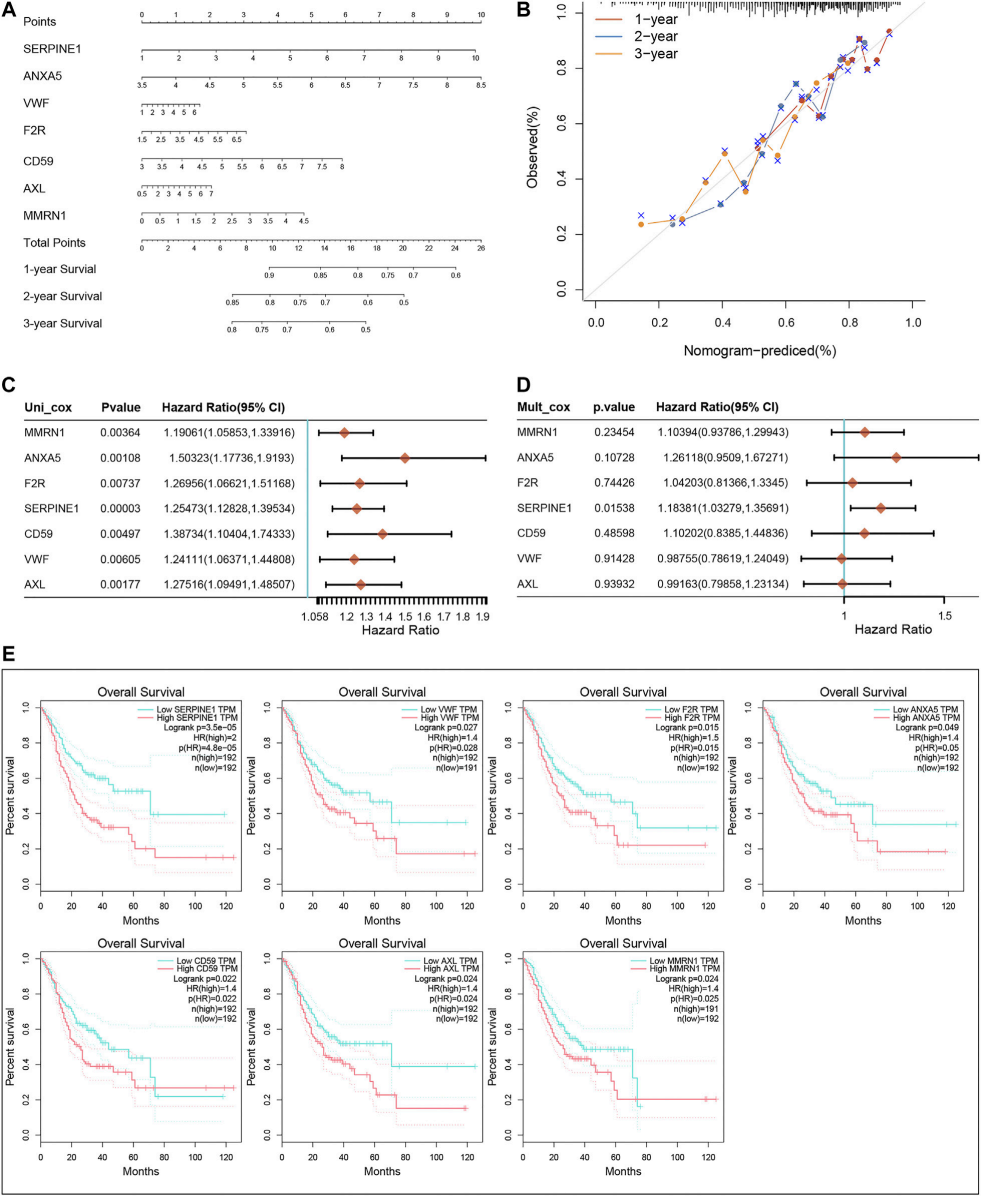
Nomogram of the Coag-Score model. **(A,B)** The nomogram and calibration curve of the Coag-Score model. **(C,D)** Univariate and multivariate Cox regression analyses for a single gene in the Coag-Score. **(E)** KM survival curves of a single gene in the Coag-Score model.

We constructed a nomogram based on the seven CRGs and Coag-Score (Figure 5A). Predictions of 1-, 2-, and 3-year survival probabilities for GC patients in the training set were shown in the calibration plot (Figure 5B). We performed univariate and multivariate Cox regression analyses for a single gene in the Coag-Score, and seven CRGs affected the prognosis of GC patients. Interestingly, the multivariate Cox regression analysis showed that SERPINE1 was an independent risk factor affecting the prognosis of GC patients (Figures 5C,D). To stabilize the results, we added age, gender, pTNM stage, and grade as covariates; SERPINE1 remained an independent risk factor for prognosis (Supplementary Figure S3A, B). In addition, we performed a survival analysis for a single gene in the Coag-Score in the GEPIA online database (Figure 5E), and the OS of patients in the high expression group was significantly lower than that of those in the low expression group.

### Analysis of enriched pathways between high-risk and low-risk cohorts

**FIGURE 6:**
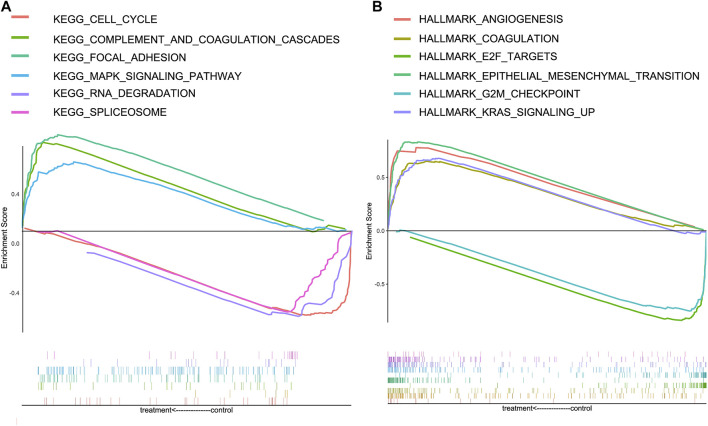
Gene Set Enrichment Analysis (GSEA) for high-risk and low-risk groups in the KEGG and HALLMARK datasets. **(A)** Enrichment pathways in the KEGG dataset of high-risk and low-risk groups. **(B)** Enrichment pathways in HALLMARK dataset of high-risk and low-risk groups.

To further explore the underlying mechanism of CRGs, HALLMARK and KEGG gene sets were analyzed between the high-risk and low-risk groups; the positive and negative correlation pathways are shown in Table 1. The genes in the high-risk group were enriched in focal adhesion, MAPK signaling pathway, complement and coagulation cascades, angiogenesis, coagulation, epithelial–mesenchymal transition, and KRAS signaling pathway. Low-risk group genes are enriched in RNA degradation, spliceosome, cell cycle, E2F targets, and G2M checkpoint (Figure 6).

**TABLE 1:** Result of Gene Set Enrichment Analysis (GSEA) between high-risk and low-risk groups.

Name	ES	NES	*p*-value
FOCAL_ADHESION	0.76913476	2.37036	0.001
MAPK_SIGNALING_PATHWAY	0.5525822	2.2472603	0.006
KRAS_SIGNALING_UP	0.6791901	2.1400163	0
ANGIOGENESIS	0.7791254	1.7455677	0.012145749
COAGULATION	0.651279	2.2354453	0
COMPLEMENT_AND_COAGULATION_CASCADES	0.7084431	2.2053964	0.012
EPITHELIAL_MESENCHYMAL_TRANSITION	0.8335778	2.0261042	0
RNA_DEGRADATION	−0.69585156	−2.1742415	0.018
SPLICEOSOME	−0.6367163	−2.092218	0.044
CELL_CYCLE	−0.67737764	−1.8732674	0.22
G2M_CHECKPOINT	−0.7602773	−1.9721214	0.002070393
E2F_TARGETS	−0.84644043	−1.9255103	0

### Analysis of tumor immune cell infiltration and immune checkpoint gene expression levels

**FIGURE 7:**
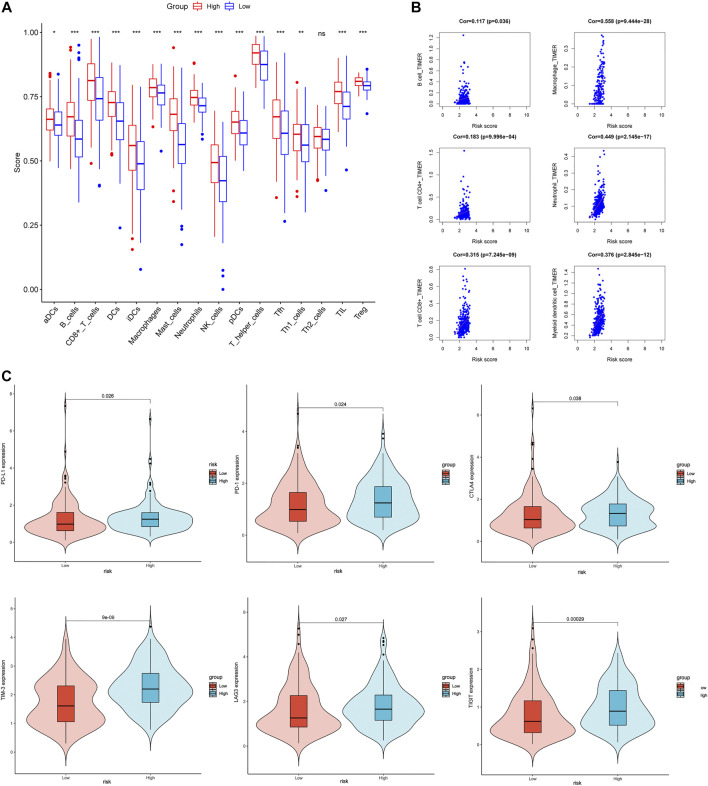
Correlation between Coag-Score and tumor immune infiltrating cells and immune checkpoints. **(A)** The ssGSEA scores between the high-risk and low-risk groups in the TCGA cohort. **(B)** Correlation between Coag-Score and immune infiltrating cells in the TIMER database. **(C)** Levels of immune checkpoint gene expression in high-risk and low-risk groups in the TCGA cohort.

In order to investigate the relationship between Coag-Score and tumor immune cell infiltration, ssGSEA was performed in the TCGA cohort. Fifteen immune cells had significantly higher infiltration enrichment fractions in the high-risk group: aDCs, B cells, CD8^+^ T cells, dendritic cells (DCs), immature dendritic cells (iDCs), mast cells, neutrophils, NK cells, plasmacytoid dendritic cells (pDCs), T helper cells, follicular helper T cells (Tfh), helper T cells 1 (Th1 cells), helper T cells 2 (Th2 cells), tumor infiltrating lymphocytes (TIL), and regulatory T cells (Tregs) (Figure 7A). We further analyzed the Coag-Score in the TIMER database, and we found that the Coag-Score was positively correlated with B cell, T-cell CD4^+^, T-cell CD8^+^, neutrophil, and myeloid dendritic cell (Figure 7B). In addition, we compared the expression of several critical immune checkpoints between high-risk and low-risk groups. The expression levels of PD-L1, PD-1, CTLA-4, TIM-3, LAG-3, and TIGIT were significantly increased (Figure 7C), indicating that patients in the high-risk group may respond better to immune checkpoint inhibitors.

### Validation of CRG expression in GC tissues

**FIGURE 8:**
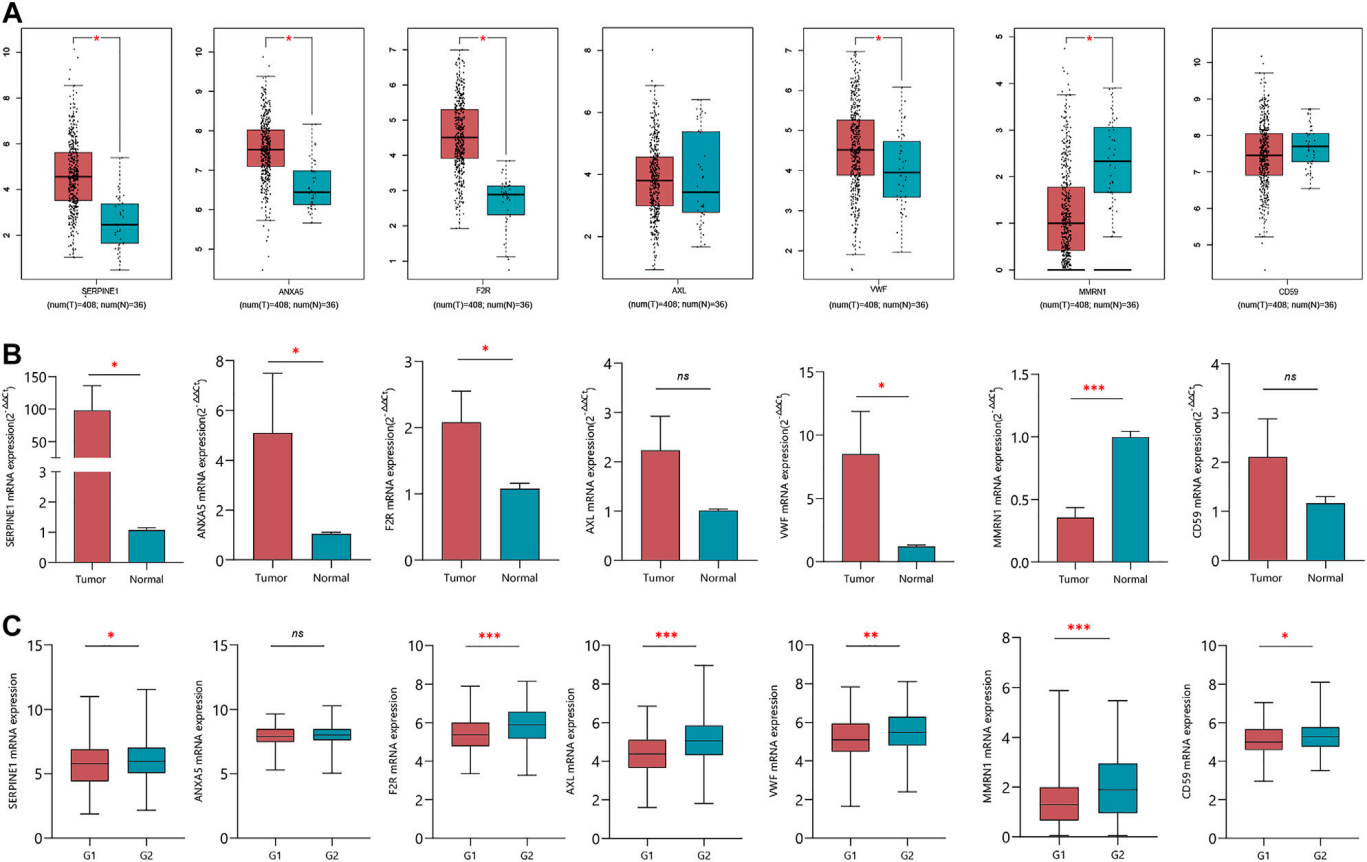
Verification of the expression of seven CRGs in normal and tumor tissues. **(A)** mRNA expression of seven CRGs in GEPIA online tool. **(B)** qRT-PCR verified the expression of seven CRGs in 10 pairs of GC clinical samples. **(C)** mRNA expression of seven CRGs in G1 and G2 groups.

**FIGURE 9:**
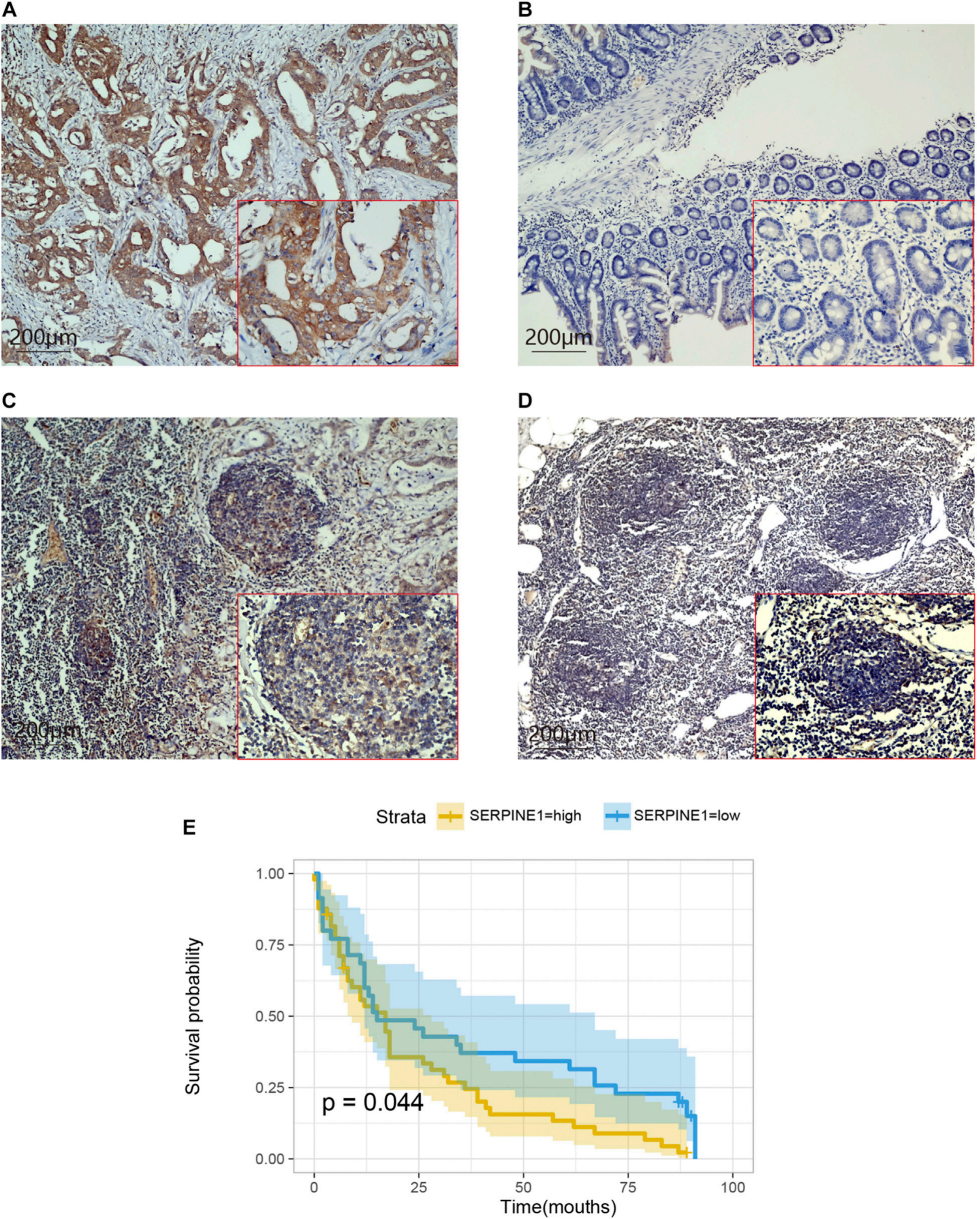
Expression and prognostic value of SERPINE1 in GC. **(A)** Positive staining of SERPINE1 in GC tissues. **(B)** Negative staining of SERPINE1 in adjacent tissues. **(C)** Positive staining of SERPINE1 in positive lymph node metastasis tissues. **(D)** Negative staining of SERPINE1 in negative lymph node metastasis tissues. **(E)** The survival analysis SERPINE1 low and high expression groups.

We verified the expression levels of seven CRGs from mRNA levels. The GEPIA results showed that SERPINE1, ANXA5, F2R, and VWF increased significantly in tumor tissue. Although AXL was not statistically significant, there was a trend of high expression in tumor tissue. While MMRN1 was lowly expressed in tumor tissues, there was no significant difference in the expression level of CD59 (Figure 8A). In 10 pairs of GC samples, we performed qRT-PCR experiments, and the results showed that the mRNA expression of SERPINE1, ANXA5, F2R, and VWF was significantly higher than that in adjacent tissue. MMRN1 was expressed low in tumor tissue. Although there was no difference in AXL and CD59 mRNA in paired adjacent tissue and cancer tissue (Figure 8B), we analyzed the expression of seven CRGs in different pathological grades in the TCGA database. We integrated the pathological grades G1 and G2 as Group 1 (G1) and G3 as Group (G2). The results showed that the mRNA expression levels of the CRGs increased significantly in G2 compared to G1, except for ANXA5 (Figure 8C). But the mRNA expression of ANXA5 increased significantly in IV stage compared to II and III stages in the TCGA database (Supplementary Figure S4). The multivariate Cox regression analysis showed that SERPINE1 was an independent risk factor affecting the prognosis of GC patients. We used GC tissue samples from our center to verify the expression of SERPINE1 in GC and its prognostic value by IHC. In 84 cases, the expression of SERPINE1 was significantly increased in GC tissues compared with adjacent tissues (Figures 9A,B). Moreover, SERPINE1 was positively expressed in tissues with positive lymph node metastasis (Figures 9C,D). In survival analysis, the SERPINE1 high expression group had a worse prognosis than the low SERPINE1 expression group (Figure 9E).

## Discussion

The morbidity and mortality of GC are increasing year by year. Despite the continuous improvement in therapeutic drugs and treatment methods, once GC recurs or metastasizes, the 5-year survival rate of patients decreases significantly (Guggenheim and Shah, 2013). Therefore, identifying effective prognostic biomarkers is crucial for predicting the occurrence and controlling disease progression in GC. There has been a great deal of interest in understanding tumor-related molecular pathways, with a focus on finding biomarkers associated with early diagnosis of cancer, tumor progression, chemotherapy, targeting, and immunotherapy responses, and influencing overall survival. Some of these studies have focused on characterizing genes and proteins associated with coagulation and fibrinolytic systems in carcinogenesis (Singh and Malviya, 2022).

Coagulation is a dynamic system in which the balance between coagulation and bleeding is always maintained in normal physiological conditions and often changes in disease conditions (Palta et al., 2014). Since 1960, hyperfibrinogen and hypercoagulability have been associated with rapidly growing tumors (Brugarolas and Elias, 1973). Systemic activation of hemostasis and thrombosis has been exhaustively implicated in cancer pathogenesis, progression, and metastasis (Langer and Bokemeyer, 2012; Lima and Monteiro, 2013). Disturbances of the coagulation system occur in GC. VTE is responsible for 10–20% of GC deaths. The incidence of clinically related VTE in GC patients was >5% in the first year after diagnosis and reached 12–17% in late GC, and the 2-year cumulative incidence of VTE in advanced GC increased to 24.4% (Lee et al., 2010; Larsen et al., 2015). The coagulation system is pivotal as a reservoir of tumor response markers and tumor angiogenesis, and the development of more effective antiangiogenic drugs.

We focused on the relationship between coagulation-related genes and the prognosis of GC, screened genes related to the clinical characteristics of GC by WGCNA, and then constructed a risk model of seven CRGs by LASSO regression. SERPINE1 is a member of the serine superfamily and encodes plasminogen activation inhibitor 1 (PAI-1). PAI-1 inhibits fibrinolysis and regulates plasminogen-induced extracellular matrix degradation and signal transduction by binding to the serine active center of uPA and tPA, resulting in a loss of plasminogen activity (Sprengers and Kluft, 1987). Many studies have found that SERPINE1 is abnormally expressed in GC tissue through bioinformatics analysis, and the expression level of SERPINE1 is negatively correlated with the prognosis of GC (Yang et al., 2019; Meng et al., 2020; Nie et al., 2020; Wu et al., 2020). Sakakibara found that the level of SERPINE1 increased significantly with the increase in tumor stage, leading to the occurrence of the malignant phenotype of tumors (Sakakibara et al., 2008). Downregulation of SERPINE1 can effectively reduce peritoneal metastasis and tumor progression in GC (Nishioka et al., 2012). SERPINE1 overexpression promotes malignant progression and poor prognosis in GC (Chen et al., 2022). ANXA5 is an anticoagulant protein that acts as an indirect inhibitor of the thromboplastin-specific complex, which is involved in the blood coagulation cascade (Ravassa et al., 2005). ANXA5 is a calcium-dependent lipid-binding protein secreted in the extracellular matrix (Bauwens, 2016), commonly used to detect apoptosis, drug transport, or as an adjunct to chemotherapy because of its high affinity for phosphatidylserine (PS) binding ability (Gerke and Moss, 2002). ANXA5 played a role in developing ovarian cancer, cervical cancer, and colorectal cancer. It was considered a diagnostic and prognostic marker (Xue et al., 2009; Li et al., 2012; Hassan et al., 2018). However, studies on ANXA5 in GC are limited, and the prognostic value of ANXA5 in GC is unclear. Wang et al. showed that ANXA5 might act as an anticancer protein, inhibit cell proliferation and metastasis, and promote cell apoptosis *via* the MEK/ERK signaling pathway (Wang et al., 2021). F2R (coagulation factor II thrombin receptor) F2R is a member of the G protein-coupled receptor family that encodes proteinase-activated receptor 1 (PAR1). High affinity receptors for activated thrombin coupled to G proteins that stimulate phosphoinositide hydrolysis may play a role in platelet activation and vascular development (Gao et al., 2020). PAR1 has been found to contribute to cell growth and invasion of tumor-derived cells (Even-Ram et al., 1998), and PAR1 is associated with poor prognosis in GC patients (Fujimoto et al., 2008). Further investigation revealed that PAR1 activation could trigger a cascade of responses that promote tumor cell growth and invasion. The activation of PAR1 leads to overexpression of NF-κB, EGFR, and TN-C, and TN-C induces EGFR activation by the autocrine mode. Therefore, PAR1 is a potentially important therapeutic target for GC (Fujimoto et al., 2010).

VWF is the largest polymeric glycoprotein in human blood. It is thought to be synthesized only in endothelial cells and megakaryocytes/platelets (Bongers et al., 2006). VWF is important in the maintenance of hemostasis; it promotes adhesion of platelets to the sites of vascular injury by forming a molecular bridge between sub-endothelial collagen matrix and platelet–surface receptor complex GPIb-IX-V. It also acts as a chaperone for coagulation factor VIII, delivering it to the site of injury, stabilizing its heterodimeric structure, and protecting it from premature clearance from plasma (Pagliari et al., 2021). VWF as a primary platelet ligand has been widely used as a biomarker for cancer and associated inflammation. Cancer-derived VWF enhances gastric adenocarcinoma metastasis through experiments *in vivo* and *in vitro* (Yang et al., 2018). Confusingly, Yin et al. (2021)found that ADAM28 from endothelial cells and GC cleaved VWF to eliminate VWF-induced apoptosis in GC cells. AXL is a member of the TYRO3, AXL, and MERTK (TAM) family of receptor tyrosine kinases (RTKs) that can be activated by the ligand Gas6, which is closely associated with tumor progression (Stitt et al., 1995). With respect to hemostasis, all three TAM receptors are located on platelets and mediate thrombogenesis and platelet stabilization. Platelet stabilization occurs after integrin activation, granule secretion, and platelet aggregation through platelet-to-platelet contact. Without this mechanism, platelet plugs disaggregate prematurely (van der Meer et al., 2014). Gas6/AXL contributes to GC cell survival and invasion by activating the Akt pathway (Sawabu et al., 2007). He et al. similarly found that the Gas6/AXL/ZEB1 axis was upregulated in GC cell lines and negatively correlated with OS in GC patients. Upregulation of ZEB1 enhanced AXL-mediated EMT, invasion, and proliferation (He et al., 2020). In platelets, MMRN1 acts as a binding protein for factor V, a key regulator of coagulation, affecting factor V function and storage (Jeimy et al., 2008). MMRN1 is secreted from platelet α-granules and Weibel–Palade bodies of endothelial cells. MMRN1 platelet-related functions include platelet adhesion, factor V regulation, and MMRN1 deficiency associated with bleeding risks in Quebec platelet disorder (Posner, 2022). Laszlo et al. (2015) made a point that MMRN1 could be used as a new marker to refine pediatric acute myeloid leukemia. Sun et al. (2020) found that MMRN1 is one of the characteristic genes associated with GC prognosis. CD59 is a glycosylphosphatidylinositol (GPI)-anchored membrane protein that regulates complement activation by inhibiting membrane attack complex (MAC) formation (Zhang et al., 2018). CD59 deficiency was identified as a pro-thrombotic factor (Tabib et al., 2018). CD59 is highly expressed in various tumor tissues and cells, such as pancreatic cancer (Bongers et al., 2006) and colorectal cancer (Watson et al., 2006). The high expression of CD59 on cancer cells can inhibit the function of complement and directly protect cells from the apoptotic cascade through various signaling pathways. Recent studies have found that CD59 has also been shown to affect tumor cell behavior and immune cell activity (Zhang et al., 2018). Kiso et al. (2002) found that CD59 expression was enhanced in intestinal metaplasia, gastric adenocarcinoma, and intestinal-type gastric cancer but not in diffuse-type GC. How CD59 functions in GC has not been further studied.

Meanwhile, we constructed a nomogram of OS in GC patients based on coagulation-related genes for the first time, and the calibration curve and C-index showed good agreement. To further explore the underlying molecular mechanisms between high-risk and low-risk groups based on Coag-Score, the GSEA was performed in the HALLMARK and KEGG pathway datasets. The results indicated that high-risk individuals were enriched in focal adhesion, MAPK signaling pathway, complement and coagulation cascades, angiogenesis, coagulation, epithelial–mesenchymal transition, and KRAS signaling pathway. Jin et al. found that celecoxib exerts anticancer effects through focal adhesion (Jin et al., 2016). The MAPK signaling pathway is widely expressed in multicellular organisms and plays a crucial role in multiple biological processes such as cell proliferation and death, differentiation, migration, and invasion. The MAPK signaling pathway is usually involved in the occurrence and progression of cancer when it is dysregulated (Yang and Huang, 2015). The KRAS oncogene plays a crucial role in tumor initiation and maintenance, and its signaling network represents a significant target for therapeutic intervention. Many inhibitors targeting kinase effectors in various Ras signaling pathways have been developed (Luo, 2021). Activation of coagulation and complement and coagulation cascades correlates with chemotherapy sensitivity and OS in cancer patients (Zhang et al., 2020). Blocking the coagulation pathway with anticoagulants and other drugs reduces the incidence of deep vein thrombosis and effectively prolongs the survival of cancer patients. EMT is one of the hallmarks of carcinogenesis and involves the redifferentiation of epithelial cells into mesenchymal cells, thereby changing the cellular phenotype to malignant cells. EMT has been shown to play a role in malignant transformation, and when it occurs in the tumor microenvironment, it significantly affects the aggressiveness of GC (Kozak et al., 2020). In conclusion, GC patients with high-risk scores are characterized by a hypercoagulable state, and therefore, according to risk scores, anticoagulation therapy for high-risk groups may improve patient outcomes.

Tumor immunotherapy stimulates the body’s immune function by increasing the immunogenicity of tumor cells and the sensitivity of effector cell killing, thereby inhibiting and killing tumor cells. The coagulation system plays an essential role in innate and adaptive immunity. Recent studies have found that the coagulation factor Xa (FXa) synthesized by monocytes and macrophages can promote tumor metastasis and immune escape by activating PAR-2. The FXa inhibitor, rivaroxaban, and PD-L1 inhibitor have synergistic anti-tumor effects (Graf et al., 2019). Elizabeth et al. found that increasing CD8 infiltration is correlated with impaired PFS and OS. Patients with higher CD8^+^ T-cell densities also have higher PD-L1 expression, indicating an adaptive immune resistance mechanism may be occurring (Thompson et al., 2017). Key gene SERPINE1 in the Coag-Score model may predict the efficacy of PD-1 antibody in patients with advanced melanoma (Ohuchi et al., 2021). We further explored the correlation between Coag-Score and immune cell infiltration. ssGSEA showed a higher enrichment score of various immune cell infiltrations in the high-risk group. TIMER analysis Coag-Score was positively correlated with CD8^+^ T cells, while PD-L1 expression was elevated in patients in the high-risk group. Therefore, high-risk patients may benefit from immunotherapy. Coag-Score may predict the efficacy of immune checkpoint inhibitors in GC patients. In addition, blockade of coagulation-related pathways may synergistically increase the effectiveness of immunotherapy.

The advantage of our study is to correlate the molecular dialogue between host cell coagulation factors and GC. We focused on coagulation-related genes to evaluate the prognostic value of GC patients. We explored the relationship between the Coag-Score model and immune infiltrating cells, and immune checkpoint expression, which provides a new predictive model and therapeutic strategy for the immunotherapy of GC. More clinical samples and further mechanistic studies are needed to verify the benefits and value of the Coag-Score model.

## Data Availability

The original contributions presented in the study are included in the article/Supplementary Material, further inquiries can be directed to the corresponding author.
