# Supplementary material for: Construction and validation of a novel coagulation-related 7-gene prognostic signature for gastric cancer
Source: Front Genet. 2022 Aug 29;13:957655. doi: 10.3389/fgene.2022.957655 (PMC9465170; doi:10.3389/fgene.2022.957655)
Supplement: Supplementary file 1 [file Table1.PDF]

Supplementary Table S1. qRT-PCR primer sequence information

Gene name	Sequence type	Primer sequence
SERPINE1	F	AACGTGGTTTTCTCACCCTAT
	R	CAATCTTGAATCCCATAGCTGC
VWF	F	CATGAAGCCATCCTCACAGTAG
	R	CCTGTTACTATGACGGTGAGAT
F2R	F	TGATCATTTCCACGGTCTGTTA
	R	AAGGAATGAGTAATGCGCAATC
ANXA5	F	AACTCTTCGGAAGGCTATGAAA
	R	TGCCAAACAGAGTCTTAAAAGC
AXL	F	AGATTTATGACTATCTGCGCCA
	R	TGACATAGAGGATTTCGTCAGG
MMRN1	F	CAGCAAAGTTTGATACACACCA
	R	CTCTGCTTTTATCTTCGCTGAC
CD59	F	CTGTGGACAATCACAATGGGAATCCAAGGA
	R	GGTGTTGACTTAGGGATGAAG
